# Herbal Arsenal against Skin Ailments: A Review Supported by In Silico Molecular Docking Studies

**DOI:** 10.3390/molecules27196207

**Published:** 2022-09-21

**Authors:** Abdel Nasser B. Singab, Nada M. Mostafa, Iten M. Fawzy, Deepika Bhatia, Pooja Tanaji Suryawanshi, Atul Kabra

**Affiliations:** 1 Department of Pharmacognosy, Faculty of Pharmacy, Ain Shams University, Cairo 11566, Egypt; 2 Center of Drug Discovery and Development, Ain Shams University, Cairo 11566, Egypt; 3 Department of Pharmaceutical Chemistry, Faculty of Pharmacy, Future University in Egypt, Cairo 11835, Egypt; 4 University Institute of Pharma Sciences, Chandigarh University, Gharuan, Mohali 140413, Punjab, India

**Keywords:** skin diseases, herbal medicine, ethnobotany, granzyme B, human leukocyte elastase, molecular docking

## Abstract

Maintaining healthy skin is important for a healthy body. At present, skin diseases are numerous, representing a major health problem affecting all ages from neonates to the elderly worldwide. Many people may develop diseases that affect the skin, including cancer, herpes, and cellulitis. Long-term conventional treatment creates complicated disorders in vital organs of the body. It also imposes socioeconomic burdens on patients. Natural treatment is cheap and claimed to be safe. The use of plants is as old as mankind. Many medicinal plants and their parts are frequently used to treat these diseases, and they are also suitable raw materials for the production of new synthetic agents. A review of some plant families, viz., Fabaceae, Asteraceae, Lamiaceae, etc., used in the treatment of skin diseases is provided with their most common compounds and in silico studies that summarize the recent data that have been collected in this area.

## 1. Introduction

Molecular docking is an in silico procedure that is able to predict the mechanism of binding of a suggested ligand to its macromolecular target during the formation of a stable complex. Therefore, docking has become of great importance for the illustration of molecular interactions of natural compounds with different receptors [1,2,3].

The skin, the largest organ of the human body, functions as a physical barrier and an exterior interface of the body with the outer environment. The skin prevents the body from the invasion of external pathogens, as well as mechanical, thermal, and physical injuries from any substance that can be hazardous to humans. Just like any other organ and system of the body, this system is also very complex. The skin, with its derivatives such as nails, sweat glands, oil glands, and hair, makes up the integumentary system [4]. It is an incredible organ that protects the whole body. It consists of three main layers, including the epidermis (outermost layer), which consists of three types of cells, i.e., squamous cells, basal cells, and melanocytes; the second layer of the skin, the dermis, which contains blood and lymph vessels, hair follicles, etc.; and the subcutaneous fat layer. The focus on skin health is because everyone wants clearer, healthier, younger, and fresher skin, as skin-related complications can cause problems related to mental health, as well as low self-esteem [5].

Herbal medicine can be traced back to ancient civilizations. It entails the use of plants for medicinal purposes to cure illnesses and improve overall health [6]. Although herbal plants are low in toxicity and readily available, they play an important role in not only pharmacological research and drug production but also as plant components, being used specifically as therapeutic agents for drug synthesis [7]. The most widely used plant parts in the preparation of traditional medicines are the leaves (62%), either alone or in combination with other plant parts [6,7].

Skin disease refers to problems with the surface layer of the skin. Skin disorders have a serious impact on well-being and are difficult to manage due to their persistence [8]. Several microorganisms trigger skin ailments, including boils, scratching ringworm, skin diseases, leprosy, injury, skin infections, eczema, skin allergy inflammation, scabies, and psoriasis [9].

Scabies, a parasitic infection, has always been the most prevalent skin disorder, but, in some areas, it is entirely absent [10]. *Sarcoptes scabiei* is the mite that causes scabies. Infection with the scabies worm causes a rash of vesicles, nodules, and papules. The majority of this is due to host hypersensitivity, but the direct impact of worm invasion also plays a significant role [11].

A rash is a red, inflamed patch of skin or a set of discrete spots. Irritation, inflammation and allergies, fundamental conditions, and structural issues may all contribute to these symptoms. Acne, eczema, psoriasis, hives, etc., are causes of rashes [4].

Atopic eczema, a chronic condition that affects people who are genetically organized to overreact towards environmental stimuli, has become an inflammatory disease. It is often seen in people with asthma, allergic rhinitis, and atopy symptoms. Eczema is a common skin problem in children. Severe skin dryness and inflammation, scaly patches, redness, and lichenified plaque with abrasions are the most common dermatitis symptoms [12].

Acne is a contagious disease and one of the most common in humans. Acne leads to seborrhea, papules, comedowns, blackheads, nodules, and scars [13]. Acne is most often found on the face, chest area, and back of people who have a large number of oil glands [14].

Psoriasis is an inflammatory skin problem that causes keratinocytes, excessive proliferation resulting in scaly patches, extreme inflammation, and erythema [15].

The uncontrolled development of cells present in the skin is known as skin cancer. It occurs due to unfixed DNA damage to skin cells, most commonly due to UV from sunlight, causing mutations and even genetic abnormalities. This causes skin cells to grow rapidly, resulting in the formation of malignant tumors [16].

A burn is considered tissue damage due to fire, chemicals, or radiation. Burn wounds are classified as superficial, partial thickness, or full thickness. Swelling, epithelization, wound contraction, and granulation are all part of the healing process after a burn wound [17].

The current review presents the effect of different medicinal plants and FDA-approved formulas on the management of various skin disorders. A molecular docking study was conducted for major components of these medicinal plants on the active sites of granzyme B and human leukocyte elastase (HLE) enzymes, aiming to identify the potential compounds or class of compounds that may be responsible for the ameliorative effects on different skin ailments.

## 2. Medicinal Plants and Skin Disorders

Medicinal plants reported for the management of skin disorders (Table 1) are classified below according to their uses.

## 3. Some Reported Mechanism of Action

The use of herbal medicine is becoming popular worldwide. Herbal medicines are preferred over synthetic medicines, as they produce fewer side effects [186,187,188,189]. Additionally, phytochemicals can treat skin ailments by different mechanisms and by displaying various biological activities such as antioxidant, anti-inflammatory, and antiallergic [190,191,192]. Each plant has its own bioactivity, which depends upon the chemical nature and potency of the constituents present in it [193,194]. Some components reduce skin inflammation by inhibiting NF-κB, for example, *Zingiber officinale*. The squeezed extract of this in rats and mice elevates TNF-α in peritoneal cells, and its long-term use can increase the level of serum corticosterone and thus reduce proinflammatory markers [195]. Drugs such as *Rosmarisum officinalis* also help in the improvement of abnormal skin conditions. It constitutes rosmarinic acid, which can disturb the system activation inhibition of the C3b attachment. It also acts on the inhibition and reduction of proinflammatory mediators such as TNF-α and IL-1 [196]. *Oenothera biennis* constitutes β-sitosterol, which modulates NO, TNF-α, IL-1β, and TXB2, leading to the suppression of COX-2 gene expression, hence causing anti-inflammatory action [197].

## 4. FDA-Approved Formulas

The Food and Drug Administration (FDA), as well as in vitro and in vivo study results, has approved bacterial cellulose (BC) and plant cellulose (PC) products to be incorporated into the biomedical field and their applications due to their biocompatibility with human cells and potential activity in wound healing and in the therapeutics field [198].

Moreover, honey, a natural product, is rich in several phenolic compounds, sugars, and enzymes that possess antioxidant, anticarcinogenic, anti-inflammatory, and antimicrobial activity. The main role of honey in the development of the wound healing process appeared to be via the acceleration of dermal repair and epithelialization, angiogenesis promotion, immune response promotion, and the reduction in healing-related infections with pathogenic microorganisms. The FDA approved many formulas containing honey as the main ingredient, among which is L-Mesitran^®^ (manufactured by Triticum Company—UK) Ointment, which consists of 48% medical-grade honey, lanolin, cod liver oil, sunflower oil, calendula, aloe vera, zinc oxide, and vitamins C and E. Additionally, Revamil Gel^®^ (manufactured by Maximed Pharrma—Lebanon) was FDA approved, containing 100% medical-grade honey, together with Therahoney^®^ Gel (manufactured by Medline Industries Inc.—USA), containing 100% Manuka honey [199].

## 5. Phytoconstituents of Medicinal Plants

Many phytochemical constituents have shown potential bioactivities, to which the biological activities of medicinal plant extracts can be attributed. Table 2 summarizes some of them in the context of treating skin disorders.

## 6. Computational Studies

### 6.1. Methodology of Molecular Docking Studies

Based on the aforementioned, human granzyme B in complex with 2-acetamido-2-deoxy-beta-D-glucopyranose [219] was downloaded from PDB (Code: 1IAU), while the crystal structure of highly glycosylated human leukocyte elastase in complex with a thiazolidinedione inhibitor (5-[[4-[[(2~{S})-4-methyl-1-oxidanylidene-1-[(2-propylphenyl)amino]pentan-2-yl]carbamoyl]phenyl]methyl]-2-oxidanylidene-1,3-thiazol-1-ium-4-olate) [220] was also downloaded from PDB (Code: 6F5M). Both enzymes were cleaned for missing amino acids or gaps in their sequences. Hydrogens were added, water molecules were removed if present, and simulation for forcefield CHARMm and partial charge MMFF was applied. A heavy atom was built, and fixation of atom constraints was applied before enzyme minimization. The receptor was identified, and the binding site was highlighted from the complexed ligand, which was later cut off for the comparative docking study. The structures of the selected active constituents were downloaded from PubChem with the .svd extension and opened in the program. A simulation for all selected 23 active constituents was applied with the CHARMm forcefield and partial charge MMFF, and ligand preparation was carried out. The 23 resulting compounds, together with the reference ligand, were allowed to dock against both enzymes using the C-docker protocol.

### 6.2. Results and Discussion of Computational Studies

Molecular docking is of great importance for illustrating the molecular interactions of natural compounds with different receptors [221]. Although each docking program operates slightly differently, they share common features that involve ligand and receptor, sampling, and scoring. Thus, a molecular docking study was performed using the selected software Discovery Studio 4.1 [222,223,224]. Twenty-three interesting phytoconstituents of the previously detailed plants were selected for in silico docking trials to explore their activity and possible mechanism of binding against two essential enzymes human granzyme B and human leukocyte elastase, where the inhibition of either or both of those enzymes could aid in the treatment of various skin diseases.

**Figure 1:**
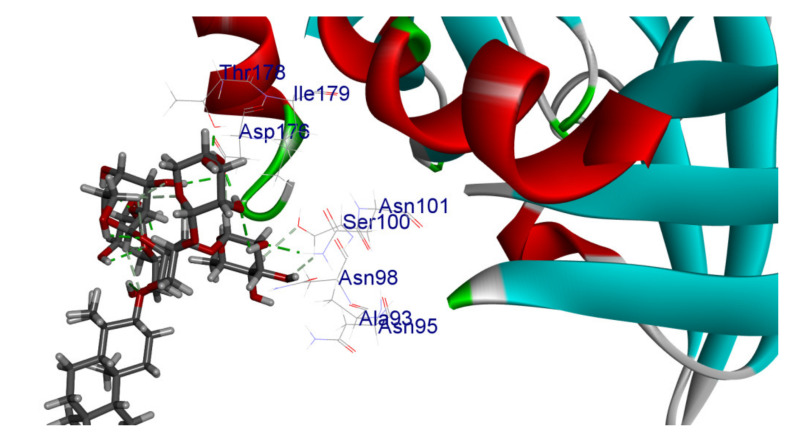
Three-dimensional (3D) interaction diagram of cyclamin (saponin) against human granzyme B (1IAU).

The 2D interaction energy of the 23 active constituents compared to the reference ligand 2-acetamido-2-deoxy-beta-D-glucopyranose, together with their C-docker interaction energy, is displayed in Table 3. The ligand displayed –27.55 Kcal/mol, saponin showed –28.10 Kcal/mol, and the rest of the constituents showed –21.42 to –1.05 Kcal/mol. Both S-methyl-L-cysteine and N-acetyl cysteine were unsuccessful in the inhibition of granzyme B. The reference ligand performed its inhibitory action via four H-bonds with essential amino acids in the granzyme B sequence (Ala 93, Asn 98, Tyr 175, and Asp 176) and via van der Waals forces with six other amino acids (Asn 95, Ser 100, Asn 101, Ser 177, Thr 178, and Ile 179). Saponin was the only constituent better than the inhibitor, displaying better interaction energy and binding mode comparable to the ligand, as shown in Figure 1. Cyclamin saponin bounded by two H-bonds with Ser 100 and three H-bonds with Asn 101, Asp 176, and Thr178, while it displayed van der Waals force attractions with Asn 93, Asn 95, Asn 98, and Ile 179.

**Figure 2:**
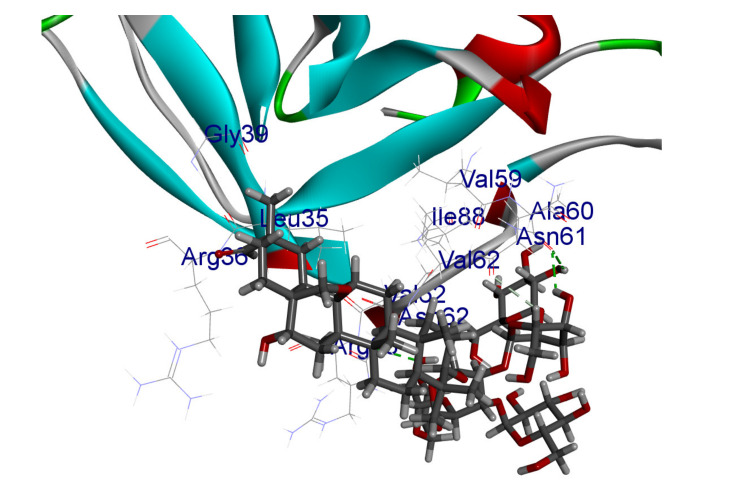
Three-dimensional (3D) interaction diagram of cyclamin (saponin) against human leukocyte elastase (6F5M).

The results of the docking study against human leukocyte elastase are presented in Table 4. It is shown that the reference complexed thiazolidinedione inhibitor displayed C-docker interaction energy equivalent to −33.57 Kcal/mol, while both constituents saponin and amaranthine displayed −48.50 and −47.62 Kcal/mol, respectively. The rest of the compounds displayed in the range of –28.97−10.60 Kcal/mol. The thiazolidinedione ligand inhibited the elastase via four essential H-bonds (Val 59, Asn 61, Asn 62A, and Val 62) and Pi-Pi bonding with Leu 35, Val 62B, and Ala 64. The van der Waals interaction was with Arg 36, Ala 60, and Ile 88. Comparably, saponin was able to inhibit elastase in the same mode, as shown in Figure 2, with better interaction energy. Cyclamin (saponin) bounded to the strategic binding site via two H-bonds with Ala 60 and two H-bonds with Asn 61 and Arg 63, Pi—Pi- bonds with Leu 35, and van der Waals interaction with Arg 36, Gly 39, His 40, Val 59, Val 62, Asn 62 Chain A, Val 62 Chain B, Ile 88, and Glu 90. On the other hand, amaranthine bounded to the binding site via three H-bonds with Ala 60, Asn 61, and Val 62, attractive charge with Arg 36, and van der Waals forces with Leu 35, Val 59, Asn 62 Chain A, and Val 62 Chain B.

Granzyme B is a serine protease found in the granules of natural killer (NK) cells and cytotoxic T cells. It is involved in inducing inflammation by cytokine release stimulation and also involved in remodeling of the extracellular matrix. Elevated levels of granzyme B are also implicated in various autoimmune diseases, several skin diseases, and type 1 diabetes [225].

On the other hand, human leukocyte elastase (HLE) is a serine proteinase involved in inflammation and tissue degradation. HLE inhibitors are believed to treat a number of diseases, such as emphysema and cystic fibrosis [220].

Natural products can have enzyme inhibitory potential for the management of different disorders [226]. According to the in silico study results, cyclamin, a saponin, is suggested to be a successful constituent for treating most underlying skin diseases owing to its chemical structure that possesses aliphatic rings, richness in oxygen atoms, and the ability to bind effectively with key amino acids of the binding sites of both granzyme B and HLE.

## 7. Conclusions

Herbs have great potential to treat various kinds of skin problems. Compared to various allopathic drugs, they have a comparatively low cost and can be of great benefit to many patients, especially poor people. Herbs are rich sources of active ingredients and can be a safer and cost-effective method for the management of skin ailments, ranging from rashes to skin cancer. FDA-approved formulas containing natural sources such as honey and biological cellulose are available and aid greatly in the treatment of skin diseases. Different mechanisms are displayed by such phytochemicals, such as inhibition of multiple inflammatory mediators, ranging from NF-κ, TNF-α, IL-1, TXB2, to COX-2. Their mechanism of action was elucidated via molecular modeling studies that were performed on the active sites of two essential proteins: granzyme B, which is a serine protease found in the granules of natural killer cells (NK cells) and cytotoxic T cells; and human leukocyte elastase (HLE), which is a serine proteinase involved in inflammation and tissue degradation. Molecular docking studies have confirmed that phytoconstituents of natural origin have potential beneficial effects on various skin disorders, especially those containing saponin. Owing to the aliphatic chains and structure rich in oxygen atoms, cyclamin saponin was able to display a comparable and stable complex with both enzymes. C-docker interaction energy expressed by saponin was −28.10 Kcal/mol for granzyme B and −48.50 Kcal/mol for HLE. Saponin bounded to granzyme B similarly to complexed reference via two H-bonds with Ser 100 and three H-bonds with Asn 101, Asp 176, and Thr178. It displayed van der Waals force attraction with Asn 93, Asn 95, Asn 98, and Ile 179, while it bounded to the strategic binding site of HLE via two H-bonds with Ala 60 and two H-bonds with Asn 61 and Arg 63, Pi—Pi- bonds with Leu 35, and van der Waals interaction with Arg 36, Gly 39, His 40, Val 59, Val 62, Asn 62 Chain A, Val 62 Chain B, Ile 88, and Glu 90.

## Figures and Tables

**Table 1:** Botanical sources and medicinal plants used to treat different skin disorders.

No.	Botanical Source (Latin Name, Common Name, Family)	Uses	References
A	Medicinal Plants Used to Treat Skin infections		
1	*Achyranthes aspera*	Used to treat boils and scabies	[18]
	Prickly chaff flower		
	Family Amaranthaceae		
2	*Aconitum chasmanthum*	Used to treat mumps and measles	[19]
	Gaping monkshood		
	Family Ranunculaceae		
3	*Butea monosperma*	Used to treat skin diseases such as inflammation	[20]
	Flame of forest		
	Family Fabaceae		
4	*Boerhavia diffusa*	Used to treat abscesses	[21]
	Tar vine, wine flower		
	Family Nyctaginaceae		
5	*Curcuma longa*	Used to treat skin inflammation	[22]
	Turmeric		
	Family Zingiberaceae		
6	*Crocus sativus*	Used to treat psoriasis	[23]
	saffron		
	Family Iridaceae		
7	*Commelina benghalensis*	Used to treat wound infection	[24]
	Tropical spiderwort		
	Family Commelinaceae		
8	*Cyperus difformis*	Used to treat skin infections	[25]
	Family Cyperaceae		
9	*Cassia tora*	Used to treat psoriasis	[26]
	Stinking cassia		
	Family Caesalpiniaceae		
10	*Capsicum frutescens*	Used to treat psoriasis	[27]
	Chilli		
	Family Solanaceae		
11	*Dalbergia sissoo*	Used to treat abscesses	[28]
	North Indian rosewood		
	Family Fabaceae		
12	*Eucalyptus globulus*	Used to treat acne, fungal infections, and heal wounds	[29]
	Eucalyptus		
	Family Myrtaceae		
13	*Euphorbia wallichii*	Used to treat skin infections and warts	[30]
	Wallich spurge		
	Family Euphorbiaceae		
14	*Ficus carica*	Used to treat itching, pimples, and scabies	[31]
	Fig		
	Family Moraceae		
15	*Fagopyrum tataricum*	Used to treat erysipelas	[32]
	Tartary buckwheat		
	Family Polygonaceae		
16	*Gnaphalium affine*	Used to treat weeping pruritus of skin	[33]
	Cotton weed		
	Family Asteraceae		
17	*Juniperus excelsa*	Used to treat skin infections	[34]
	Eastern savin		
	Family Cupressaceae		
18	*Lens culinaris*	Used to treat skin infections and acne	[35]
	Lentil		
	Family Fabaceae		
19	*Marsilea quadrifolia*	Used to treat abscesses	[36]
	Water clover		
	Family Marsileaceae		
20	*Mahonia aquifolium*	Used to treat psoriasis	[37]
	Oregon grape		
	Family Berberidaceae		
21	*Pleurospermum brunonis*	Used to treat skin infections	[38]
	Brown’s paper cup flower		
	Family Apiaceae		
22	*Pinus roxburghii*	Used to treat pruritus, inflammation, and other skin diseases	[39]
	Chir pine		
	Family Pinaceae		
23	*Pinus wallichiana*	Used to treat wound infection	[40]
	Bhutan pine		
	Family Pinaceae		
24	*Rubia cordifolia*	Used to treat psoriasis	[41]
	Common madder		
	Family Rubiaceae		
25	*Solanum nigrum*	Used to treat pimples, pustules, ringworms, eczema, syphilitic ulcers, and leukoderma	[42,43]
	Black nightshade		
	Family Solanaceae		
26	*Simmondsia chinensis*	Used to treat acne and psoriasis	[44]
	Jojoba		
	Family Buxaceae		
27	*Taxus wallichiana*	Used to treat psoriasis and ringworm	[45]
	Himalayan yew		
	Family Taxaceae		
28	*Tectona grandis*	Used to treat pruritus and heal wounds	[46,47]
	Teak		
	Family Lamiaceae		
29	*Thespesia populne*	Used to treat psoriasis	[48]
	Indian tulip tree		
	Family Malvaceae		
30	*Wrightia tinctoria*	Used to treat psoriasis	[49]
	Sweet indrajao		
	Family Apocynaceae		
**B**	**Medicinal Plants Used to Treat Eczema**		
31	*Abrus precatorious*	Used to treat eczema	[50]
	Rosary pea		
	Family Fabaceae		
32	*Avena sativa*	Used to treat eczema, wounds, inflammation, itching, burns, and irritation	[51]
	Oat		
	Family Poaceae		
33	*Arnebia euchroma*	Used to treat burns, eczema, and dermatitis	[52,53]
	Pink arnebia		
	Family Boraginaceae		
34	*Actinidia deliciosa*	Used to treat inflammation and eczema	[54]
	Kiwi fruit		
	Family Actinidiaceae		
35	*Aristolochia indica*	Used to treat eczema and wounds	[55]
	Indian birthwort		
	Family Aristolochiaceae		
36	*Betula alba*	Used to treat eczema, psoriasis, and acne	[56]
	Paper birch		
	Family Betulaceae		
37	*Cannabis sativus*	Used to treat sores, eczema, dermatitis, psoriasis, seborrheic, and lichen planus	[57]
	Charas, ganja		
	Family Cannabaceae		
38	*Matricaria chamomilla*	Used to treat eczema and skin inflammation	[58,59]
	Chamomile		
	Family Asteraceae		
39	*Sarco asoca*	Used to treat skin diseases, inflammation, eczema, and scabies	[60]
	Ashoka		
	Family Caesalpiniaceae		
40	*Saponaria officinalis*	Used to treat eczema, acne, boils, and psoriasis	[61,62]
	soapworts		
	Family Caryophyllaceae		
41	*Vitex negundo*	Used to treat skin diseases such as eczema, acne, pimples, ringworms, etc.	[35]
	Nirgundi		
	Family Verbenaceae		
**C**	**Medicinal Plants Used for Wound healing**		
42	*Achillea millefolium*	Used to treat burn wounds	[63]
	Common Yarrow		
	Family Asteraceae		
43	*Albizia lebbeck*	Used for wound healing, leucoderma, itching, and inflammation	[64]
	Siris		
	Family Fabaceae		
44	*Allium sativum*	Used to treat psoriasis, scars, and heal wounds	[65]
	Garlic		
	Family Alliaceae		
45	*Aloe barbadensis*	Used to treat skin injuries	[66]
	Aloe vera		
	Family Aloeaceae		
46	*Alternanthera brasiliana*	Used to heal inflammation wounds	[64]
	Brazilian joyweed		
	Family Amaranthaceae		
47	*Abelmoschus esculentus*	Used to cure pimples and wounds	[67]
	Okra		
	Family Malvaceae		
48	*Adiantum venustum D*	Used to heal wounds	[68]
	Himalayan maidenhair		
	Family Pteridaceae		
49	*Argemone Mexicana*	Used to treat wounds	[69]
	Mexican poppy		
	Family Papaveraceae		
50	*Alkanna tinctoria*	Used to treat itching, skin wounds, and rashes	[70]
	Alkanet		
	Family Boraginaceae		
51	*Brassica oleracea*	Used to treat dermatitis and wounds	[71]
	Red cabbage		
	Family Brassicaceae		
52	*Berberis lycium*	Used to heal wounds	[72]
	Indian lycium		
	Family Berberidaceae		
53	*Bergenia ciliata*	Used to heal wounds	[73,74]
	Winter begonia		
	Family Saxifragaceae		
54	*Bergenia ligulata*	Used to heal wounds and treat boils	[75]
	Asmabhedaka		
	Family Saxifragaceae		
55	*Bauhinia purpurea*	Used to heal wounds and treat inflammation	[76]
	Orchid tree		
	Family Fabaceae		
56	*Carissa spinarum*	Used to heal wounds and treat boils	[77]
	Bush plum		
	Family Apocynaceae		
57	*Cannabis sativa*	Used to treat dandruff and heal wounds	[78]
	Marijuana, hemp		
	Family Cannabaceae		
58	*Capparis decidua*	Used to heal wounds	[79]
	Bare caper		
	Family Capparaceae		
59	*Cynodon dactylon*	Used to heal wounds and skin problems	[80,81]
	Bermuda grass		
	Family Poaceae		
60	*Cocos nucifera*	Used to treat skin wounds	[82]
	Coconut		
	Family Arecaceae		
61	*Euphorbia helioscopia*	Used to heal wounds	[83,84]
	Sun spurge		
	Family Euphorbiaceae		
62	*Ferula foetida*	Used to heal wounds	[85]
	Asafoetida, Hing		
	Family Apiaceae		
63	*Ficus benghalensis*	Used to treat skin injuries	[86]
	Banyan tree		
	Family Moraceae		
64	*Gerbera gossypina*	Used to heal wounds	[87]
	Hairy gerbera daisy		
	Family Asteraceae		
65	*Galium aparine*	Used to treat wounds as an antiseptic	[88]
	Goosegrass		
	Family Rubiaceae		
66	*Hackelia americana*	Used to treat wounds, tumors, and inflammation	[89]
	Nodding stickseed		
	Family Boraginaceae		
67	*Hypericum perforatum*	Used to treat wounds, abrasions, inflammatory skin disease, and burns	[90]
	Perforatejohn’s wort		
	Family Hypericaceae		
68	*Isodon rugosus*	Used to heal wounds	[91]
	Wrinkled leaf isodon		
	Family Lamiaceae		
69	*Launaea nudicaulis*	Used to heal wounds	[92]
	Bhatal		
	Family Asteraceae		
70	*Momordica charantia*	Used to heal wounds	[93]
	Bitter gourd		
	Family Cucurbitaceae		
71	*Micromeria biflora*	Used to heal wounds and treat skin infections	[94]
	Lemon savory		
	Family Lamiaceae		
72	*Nigella sativa*	Used to heal wounds	[95,96]
	Black cumin		
	Family Ranunculaceae		
73	*Plantago major*	Used to treat wounds	[97]
	Great plantain		
	Family Plantaginaceae		
74	*Plantago lanceolata*	Used to heal wounds	[98]
	Ribwort plantain		
	Family Plantaginaceae		
75	*Rumex dissectus*	Used to stop wound bleeding	[99]
	Arrowleaf dock		
	Family Polygonaceae		
76	*Salvia moorcroftiana*	Used to treat skin itching and wound healing	[100]
	Kashmir salvia		
	Family Lamiaceae		
77	*Trigonella foenum-graecum*	Used to heal wounds	[101,102]
	Fenugreek		
	Family Fabaceae		
78	*Tephrosia purpurea*	Used to heal wounds	[103]
	Wild indigo		
	Family Fabaceae		
79	*Urtica dioica*	Used to heal wounds	[104,105]
	Stinging nettle		
	Family Urticaceae		
80	*Verbascum Thapsus*	Used to treat pimples, heal wounds, and treat other skin problems	[106]
	Common mullein		
	Family Scrophulariaceae		
**D**	**Medicinal Plants Used to Treat Skin Burns**		
81	*Astilbe thunbergii*	Used to treat burns	[107]
	Astilbe		
	Family Saxifragaceae		
82	*Anaphalis margaritacea*	Used to treat sunburn	[108]
	Pearly everlasting		
	Family Asteraceae		
83	*Aquilegia pubiflora*	Used to heal wounds and treat skin burns	[109]
	Himalayan columbine		
	Family Ranunculaceae		
84	*Amygdalus communis*	Used to treat burn wounds	[53]
	Almonds		
	Family Rosaceae		
85	*Bergenia stracheyi*	Used to treat sunstroke and heal wounds	[110]
	Himalayan Bergenia		
	Family Saxifragaceae		
86	*Calendula officinalis*	Used to treat burns and bruises	[111]
	Marigold		
	Family Asteraceae		
87	*Cucumis melo*	Used to treat skin burns	[112]
	Muskmelon		
	Family Cucurbitaceae		
88	*Corydalis govaniana*	Used to treat skin burns	[113]
	Govan’s corydalis		
	Family Papaveraceae		
89	*Carica candamarcensis*	Used to treat burn wounds	[114]
	Mountain papaya		
	Family Caricaceae		
90	*Clitoria ternatea*	Used to treat boils, acne, and skin outbreaks	[115]
	Butterfly pea		
	Family Fabaceae		
91	*Datura stramonium*	Used to treat boils	[116]
	Jimsonweed, thornapple		
	Family Solanaceae		
92	*Dodonaea viscosa*	Used to treat skin burns and heal wounds, acne, pimples, rashes, itching, and pustules	[117,118,119]
	Hop bush		
	Family Sapindaceae		
93	*Echinacea angustifolia*	Used to treat psoriasis, burns, acne, ulcers, and skin wounds	[120]
	Purple coneflower		
	Family Asteraceae		
94	*Ginkgo biloba*	Used to treat skin burns	[121]
	Maidenhair tree		
	Family Ginkgoaceae		
95	*Hippophae rhamnoides*	Used to treat rashes and skin burns	[122,123]
	Sea buckthorn		
	Family Elaeagnaceae		
96	*Impatiens edgeworthii*	Used to treat skin burns	[124]
	Edgeworth Balsam		
	Family Balsaminaceae		
97	*Mangifera indica*	Protect skin from sun damage	[125]
	Mango		
	Family Anacardiaceae		
98	*Malus pumila*	Used to treat boils	[126]
	Apple		
	Family Rosaceae		
99	*Malva sylvestris*	Used to treat burn wounds	[53]
	High mallow		
	Family Malvaceae		
100	*Matricaria chamomilla*	Used to treat burn wounds	[127]
	Chamomile		
	Family Asteraceae		
101	*Onosma hispida*	Used to treat skin burns	[128]
	Bristly onosma		
	Family Boraginaceae		
102	*Portulaca oleracea*	Used to treat burns, skin eruptions, rashes, skin inflammation, eczema, abscesses, and pruritus	[129,130,131]
	Purslane, little hogweed		
	Family Portulacaceae		
103	*Pisum sativum*	Used to treat skin burns	[132]
	Garden pea		
	Family Fabaceae		
104	*Picrorhiza kurroa*	Used to treat burning sensation	[133]
	Kutki		
	Family Plantaginaceae		
105	*Rumex dentatus*	Used to treat boils	[134]
	Toothed dock		
	Family Polygonaceae		
106	*Rubus abchaziensis*	Used to treat boils and wounds	[135]
	Akhray		
	Family Rosaceae		
107	*Solanum virginianum*	Used to treat swelling of skin	[136]
	Thorny nightshade		
	Family Solanaceae		
108	*Scrophularia deserti*	Used to treat burn wounds	[53]
	Desert figwort		
	Family Scrophulariaceae		
109	*Sesamum indicum*	Used to treat burn wounds	[137]
	Sesame		
	Family Pedaliaceae		
110	*Silybum marianum*	Used to treat burn wounds and improve skin health	[138]
	Blessed thistle		
	Family Asteraceae		
111	*Tamarix aphylla*	Used to treat skin burns and wounds	[139]
	Athel		
	Family Tamaricaceae		
112	*Tridax procumbens*	Used to treat burn wounds	[140]
	Coatbuttons, tridax daisy		
	Family Asteraceae		
113	*Zanthoxylum armatum*	Used to treat skin burns	[141]
	Winged prickly ash		
	Family Rutaceae		
**E**	**Medicinal Plants Used to Treat Miscellaneous Disorders**		
114	*Allium cepa*	Used to treat skin lesions	[142]
	Garden onion		
	Family Alliaceae		
115	*Azadirachta indica*	Used to treat acne and protect skin from UV rays	[143]
	Neem		
	Family Meliaceae		
116	*Anethum graveolens*	Used to treat pimples	[144]
	Dill		
	Family Apiaceae		
117	*Androsace rotundifolia lehm.*	Used to treat skin problems	[145]
	Rock jasmine		
	Family Primulaceae		
118	*Arnica montana*	Used as anti-inflammatory to treat boils and acne eruptions	[146,147]
	Mountain arnica		
	Family Asteraceae		
119	*Bauhinia variegata*	Used to treat skin disease and skin ulcers	[148]
	Kachnar, orchid tree		
	Family Fabaceae		
120	*Beta vulgaris*	Used to treat tumors	[149]
	Beetroot		
	Family Brassicaceae		
121	*Brassica juncea*	Used against skin eruptions and ulcers	[150,151]
	Mustard		
	Family Brassicaceae		
122	*Berberis aquifolium*	Used to treat acne scars	[152]
	Oregon grape		
	Family Berberidaceae		
123	*Camellia sinensis*	Used to treat skin tumors and cancer	[153]
	Green Tea		
	Family Theaceae		
124	*Coriandrum sativum*	Used to treat pimples	[154,155]
	Dhaniya		
	Family Apiaceae		
125	*Calotropis procera*	Used to treat inflammation	[156]
	Giant milkweed		
	Family Apocynaceae		
126	*Cerastium fontanum*	Used to treat skin diseases; also acts as anti-inflammatory	[157]
	Mouse ear chickweed		
	Family Caryophyllaceae		
127	*Citrus medica*	Used to treat skin irritation	[158,159]
	Citron		
	Family Rutaceae		
128	*Citrus sinensis*	Used to treat pimples	[160]
	orange		
	Family Rutaceae		
129	*Catharanthus roseus*	Used to cure pimples	[161]
	Periwinkle		
	Family Apocynaceae		
130	*Carthamus tinctorius*	Used to treat eruptive skin problems	[162]
	safflower		
	Family Asteraceae		
131	*Clerodendrum viscosum*	Used as antiseptic skin wash	[163]
	Hill glory bower		
	Family Verbenaceae		
132	*Equisetum arvense*	Used to treat skin allergy	[164]
	Field horsetail		
	Family Equisetaceae		
133	*Lavendula officinalis*	Used to prevent and heal acne	[165]
	Lavender		
	Family Labiatae		
134	*Lawsonia inermis*	Used to treat inflammation and tumors	[166]
	Henna		
	Family Lythraceae		
135	*Lycopersicon esculentum*	Used to treat acne and sunburn	[167]
	Tomato		
	Family Solanaceae		
136	*Ledum groenlandicum oedar*	Used to treat itching, acne, and redness	[61]
	Labrador tea		
	Family Ericaceae		
137	*Mirabilis jalapa*	Used to treat allergic skin disorders	[168]
	Four o’clock		
	Family Nyctaginaceae		
138	*Melia azedarach*	Used to treat pimples and inflammation	[169]
	Persian lilac		
	Family Meliaceae		
139	*Myrsine Africana*	Used to treat skin disorders	[170]
	Cape myrtle		
	Family Myrsinaceae		
140	*Melaleuca alternifolia*	Used to treat acne	[171]
	Tea tree		
	Family Myrtaceae		
141	*Olea europaea*	Used as skin cleanser	[172]
	Olive tree		
	Family Oleaceae		
142	*Ocimum sanctum*	Used to treat acne and inflammation	[173,174]
	Tulsi		
	Family Lamiaceae		
143	*Plumbago zeylanica*	Used to treat skin diseases such as sores, acne, and dermatitis	[31]
	Doctor bush		
	Family Plumbaginaceae		
144	*Prunus persica*	Used to treat skin disorders	[175]
	Peach		
	Family Rosaceae		
145	*Piper nigrum*	Used to treat acne	[176]
	Black pepper		
	Family Piperaceae		
146	*Pterocarpus santalinus*	Used to treat skin inflammation and acne	[177]
	Red sandalwood		
	Family Fabaceae		
147	*Rosmarinus officinalis*	Used to block skin tumor cells	[178]
	Rosemary		
	Family Lamiaceae		
148	*Ricinus communis*	Used in children for skin diseases	[179]
	Castor oil plant		
	Family Euphorbiaceae		
149	*Rheum officinale*	Used to treat acne	[180]
	Rhubarb		
	Family Polygonaceae		
150	*Salix babylonica*	Used as skin cleanser	[181]
	Weeping willow		
	Family Salicaceae		
151	*Serenoa repens*	Used to treat acne and inflammation	[182]
	Saw palmetto		
	Family Arecaceae		
152	*Thymus vulgaris*	Used to treat cellulitis	[153]
	Thyme		
	Family a		
153	*Taraxacum officinale*	Used to treat pimples	[183]
	Common dandelion		
	Family Asteraceae		
154	*Tussilago farfara*	Used to treat sores and inflammation of skin	[184]
	coltsfoot		
	Family Asteraceae		
155	*Valeriana jatamansi*	Used to treat pimples	[185]
	Jatamansi		
	Family Caprifoliaceae		

**Table 2:** Selected reported phytoconstituents of herbal plants used to treat skin diseases.

Serial No.	Botanical Name	Some Phytoconstituents and/or Classes ofCompounds	Selected Structures	Ref.
1.	*Abrus precatorious*	Stigmasterol, β-sitosterol, and abrusogenin	Abrusogenin 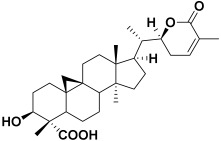	[200]
2.	*Achillea millefolium*	Chlorogenic acid, apigenin-7-glucoside, and luteolin-7-glucoside	Chlorogenic acid 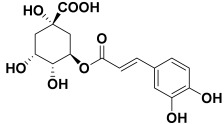	[201]
3.	*Achyranthes aspera*	Rutin, chlorogenic acid, and genistein	Genistein 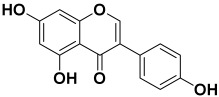	[202]
4.	*Allium cepa*	Quercetin, S-methyl-L-cysteine, cycloalliin, N-acetylcysteine, S-propyl-L-cysteine sulfoxide, and dimethyl trisulfide	Cycloalliin N-acetyl cysteine 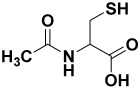 S-methyl-L-cysteine 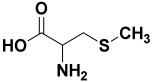	[203]
5.	*Azadirachta indica*	Nimbin, nimbanene, ascorbic acid, n-hexacosanol, nimbolide, 17-hydroxy azadiradione, 6-desacetyl nimbinene, and nimbandiol	Nimbin 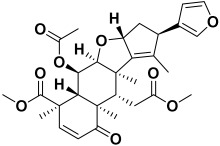	[204]
6.	*Albizia lebbeck*	Lupeol, lupenone, luteolin, rutin, sapiol, friedelin, stigmasterol, β-sitosterol, stigmasterol-3-glucoside, β-sitosterol-3-glucoside, alkaloids as 3,3-dimethyl-4-(1-aminoethyl)-azetidin-2-one, 2-amino-4-hydroxy pteridine-6-carboxylic acid, and 2,4 bis(hydroxylamino)-5-nitropyrimidine	Lupeol 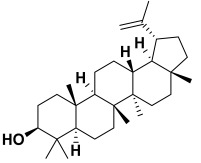	[205]
7.	*Allium sativum*	Alliin, allicin, S-allyl cysteine, diallyl sulfide, diallyl trisulfide, diallyl disulfide, and ajoene	Alliin 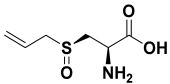	[206]
8.	*Aloe barbadensis*	Aloesin, cinnamic acid, isoaloresin D, caffeic acid, chlorogenic acid, aloin A and B, emodin, isovitexin, and orientin	Aloin 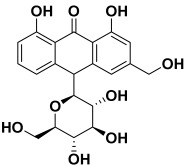	[207]
9.	*Alternanthea brasiliana*	Amaranthine, iso amaranthine, betanin, isobetanin, hydroxybenzoic acid, hydroxycinnamic acid, kaempferol glucoside, rhamnoside, and dirhamnosyl-glucoside	Amaranthine 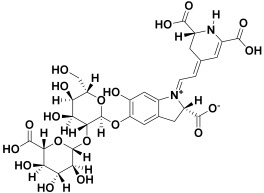	[208]
10.	*Anethumgraveolens*	Limonene, carvone, α-phellandrene, β-phellandrene, and *p*-cymene	Limonene 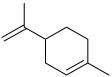	[209]
11.	*Avena sativa*	Proteins, lipids, polysaccharides, β-glycan, dietary fibers, avenanthramides, gramine alkaloid, flavonolignans, flavonoids, saponins, and sterols	Avenanthramide A 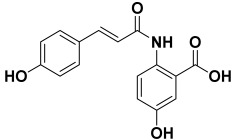	[210]
12.	*Arnebia euchroma*	Shikonin, methyllasiodiplodin, euchroquinols A-C, and 9,17-epoxy arnebinol	Shikonin, 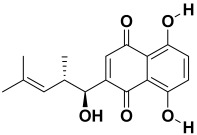	[211]
13.	*Astilbe thunbergii*	Eucryphin, astilbin, and berginin	Eucryphin 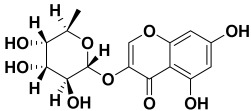	[107]
14.	*Actinidia deliciosa*	Rutin, quercitrin, quercetin, chrysin, and syringic acid	Quercetin 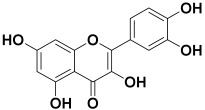	[212]
15.	*Anaphalis margaritacea*	Volatile oil contains E-caryophyllene, and its oxide, δ-cadinene, γ-cadinene, cubenol, ledol, and α-pinene	E-caryophyllene 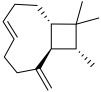	[213]
16.	*Abelmoschus esculentus*	Quercetin-3-glucoside, diglucoside, catechins, and hydroxyl cinnamic acid derivatives	Quercetin-3-glucoside 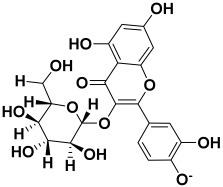	[214]
17.	*Adiantum venustum* Don	Norlupane, noroleanane, lupane triterpenoids, adiantone, and 21-hydroxyadiantone (Norhopane)triterpenes	Adiantone 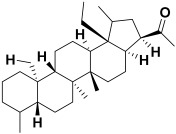	[215]
18.	*Saponaria officinalis*	Saponins	Cyclamin 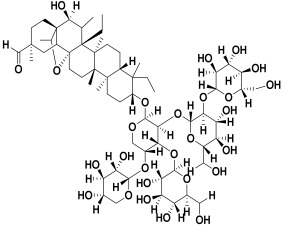	[62]
19.	*Aquilegia pubiflora*	Orientin, coumaric acid, sinapic acid, chlorogenic acid, ferulic acid, vitexin, isoorientin, and isovitexin	Orientin 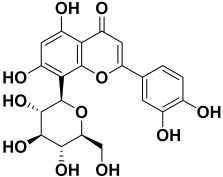	[216]
20.	*Argemone mexicana*	Berberine, oxyberberine, arginine, higenamine, pancorine, sanguinarine, β-amyrin, trans-phytol, luteolin, quercetin, quercitrin, and rutin	Berberine 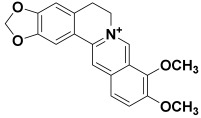	[69]
21.	*Arnica* *montana*	Sesquiterpene lactones, phenolic acids, flavonoids, helenalin, acetyl helenalin, metacryl helenalin, chlorogenic acid, 3,5-dicaffeoylquinic acid, 4,5- dicaffeoylquinic acid, quercetin-3-glucoside, quercetin-3-glucuronide, kaempferol-3-glucoside, and kaempferol-3-glucuronide	Solaniol 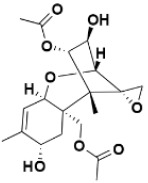	[217]
22.	*Alkanna* *tinctoria*	Alkaloid, bufadienolides, carbohydrate, flavonoids, saponins, and tannins	Bufadienolide 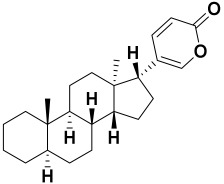	[218]

**Table 3:** Results of molecular modeling study of 24 active constituents against human granzyme B (1IAU) compared to reference complexed ligand.

Serial No.	Compound	(C-Docker Interaction Energy)	2D Interaction Diagram *	Type of Binding
1	Ligand (reference)	−27.55	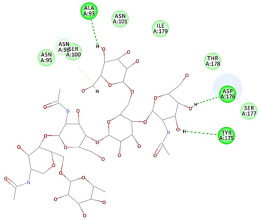	**H-bond**: Ala 93, Asn 98, Asp 176, Tyr 175 **Van der Waals**: Asn 95, Ser 100, Asn 101, Ser 177, Thr 178, Ile 179
2	Cyclamin (saponin)	−28.10	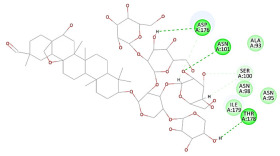	**H-bond**: Ser 100 (×2), Asn 101, Asp 176, Thr 178 **Van der Waals**: Asn 93, Asn 95, Asn 98, Ile 179
3	Amaranthine	−21.42	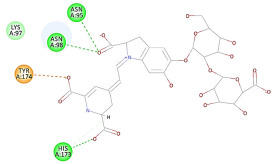	**H-bond**: Asn 95, Asn 98, His 173 (×2) **Pi-Pi**: Tyr 174 **Van der Waals**:Lys 97
4	Alliin	−18.53	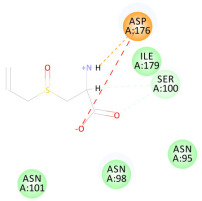	**H-bond**: Ser 100 (×2) **Pi-Pi**: Asp 176 **Van der Waals**: Asn 95, Asn 98, Asn 101, Ile 179 **Unfavorable**: Asp 176
5	Quercetin-3-glucoside	−17.59	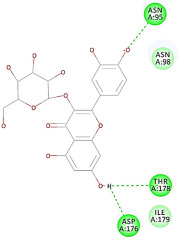	**H-bond**: Asn 95, Asp 176, Thr 178 **Van der Waals**: Asn 98, Ile 179
6	Aloin	−17.35	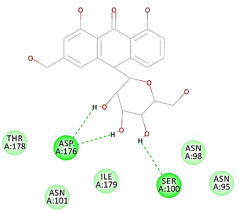	**H-bond**: Ser 100, Asp 176 (×2) **Van der Waals**: Asn 95, Asn 98, Asn 101, Thr 178, Ile 179
7	Berberine	−15.12	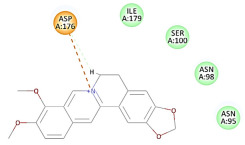	**Pi-Pi**: Asp 176 **Van der Waals**: Asn 95, Asn 98, Ser 100, Ile 179
8	Chlorogenic acid	−14.09	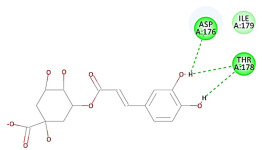	**H-bond**: Asp 176, Thr 178 (×2) **Van der Waals**: Ile 179
9	Avenanthramide A	−14.03	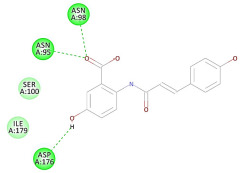	**H-bond**: Asn 95, Asn 98, Asp 176 **Van der Waals**: Ser 100, Ile 179
10	Adiantone	−12.76	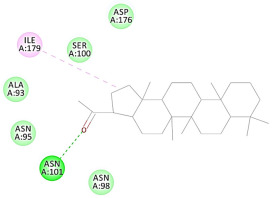	**H-bond**: Asn 101 **Pi-Alkyl**: Ile 179 **Van der Waals**: Ala 93, Asn 95, Asn 98, Ser 100, Asp 176
11	Orientin	−11.89	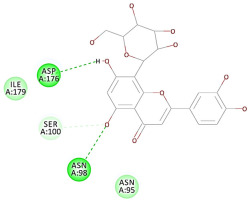	**H-bond**: Asn 98, Ser 100, Asp 176 **Van der Waals**: Asn 95, Ile 179
12	Eucryphin	−11.34	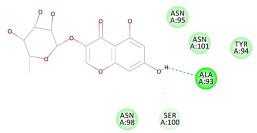	**H-bond**: Ala 93, Ser 100 **Van der Waals**: Tyr 94, Asn 95, Asn 98, Ser 100, Asn 101
13	Lupeol	−11.15	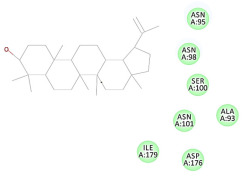	**Van der Waals**: Ala 93, Asn 95, Asn 98, Ser 100, Asn 101, Asp 176, Ile 179
14	Quercetin	−11.02	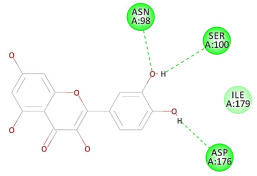	**H-bond**: Asn 98, Ser 100, Asp 176 **Van der Waals**: Ile 179
15	Abrusogenin	−10.47	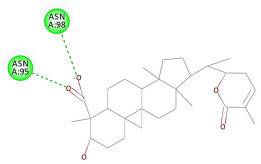	**H-bond**:Asn 95, Asn 98
16	Shikonin	−10.25	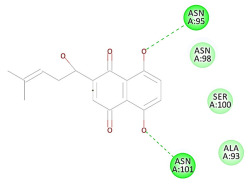	**H-bond**: Asn 95, Asn 101 **Van der Waals**: Ala 93, Asn 98, Ser 100
17	Bufadienolide	−10.05	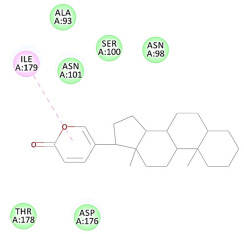	**Pi-Alkyl**: Ile 179 **Van der Waals**: Ala 93, Asn 98, Ser 100, Asn 101, Asp 176, Thr 178
18	Nimbin	−8.77	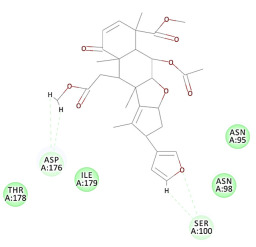	**H-bond**: Ser 100 (×2), Asp 176 (×2) **Van der Waals**: Asn 95, Asn 98, Thr 178, Ile 179
19	Genistein	−7.64	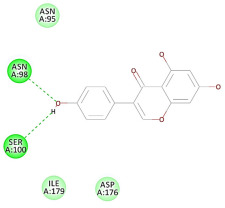	**H-bond**: Asn 98, Ser 100 **Van der Waals**: Asn 95, Asp 176, Ile 179
20	Solaniol	−7.28	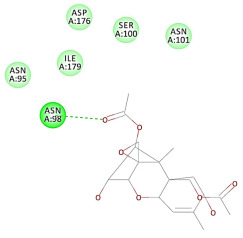	**H-bond**: Asn 98 **Van der Waals**: Asn 95, Ser 100, Asn 101, Asp 176, Ile 179
21	*E*-caryophyllene	−3.25	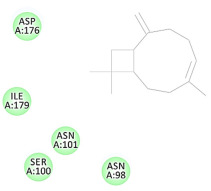	**Van der Waals**: Asn 98, Ser 100, Asn 101, Asp 176, Ile 179
22	Limonene	−2.48	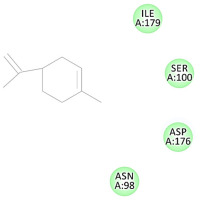	**Van der Waals**: Asn 98, Ser 100, Asp 176, Ile 179
23	S-methyl-L-cysteine	−1.79	No interaction	
24	N-acetyl cysteine	−1.05	No interaction	

* Color reference: green dotted line indicates H-bond; faint green dotted line indicates van der Waals interaction; orange dotted line indicates Pi-Pi bond; red dotted line indicates unfavorable interaction; purple dotted line indicates Pi-alkyl bond.

**Table 4:** Results of molecular modeling study of 23 active constituents against human leukocyte elastase (6F5M) compared to reference complexed ligand.

Serial No.	Compound	(C-Docker Interaction Energy)	2D Interaction Diagram *	Type of Binding
1	Ligand (reference)	−33.57	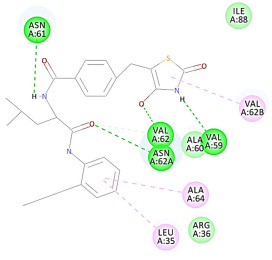	**H-bond**: Val 59, Asn 61, Asn 62A, Val 62 **Pi-Pi bond**: Leu 35, Val 62B, Ala 64 **Van der Waals**: Arg 36, Ala 60, Ile 88
2	Cyclamin (Saponin)	−48.50	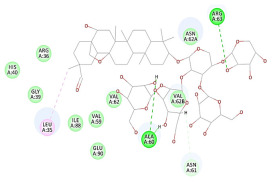	**H-bond**: Ala 60(×2), Asn 61, Arg 63 **Pi-Pi bond**: Leu 35 **Van der Waals**: Arg 36, Gly 39, His 40, Val 59, Val 62, Asn 62A, Val 62B, Ile 88, Glu 90
3	Amaranthine	−47.62	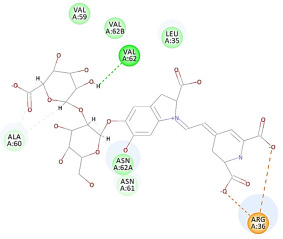	**H-bond**: Ala 60, Asn 61, Val 62 **Attractive charge**: Arg 36(×2) **Van der Waals**: Leu 35, Val 59, Asn 62A, Val 62B
4	Chlorogenic acid	−28.97	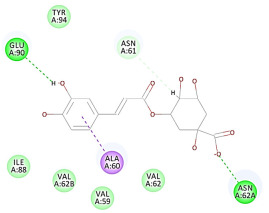	**H-bond**: Asn 61, Asn 62A, Glu 90 **Pi-sigma**: Ala 60 **Van der Waals**: Val 59, Val 62, Val 62B, Ile 88, Tyr 94
5	Quercetin-3-glucoside	−27.94	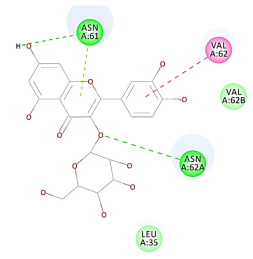	**H-bond**: Asn 61, Asn 62A **Pi-lone pair**: Asn 61 **Pi-Pi**: Val 62 **Van der Waals**: Leu 35, Val 62B
6	Orientin	−26.43	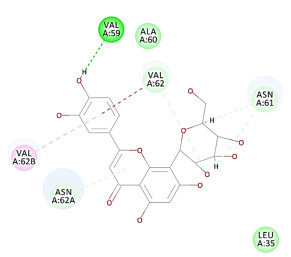	**H-bond**: Val 59, Asn 61(×2), Asn 62A, Val 62 **Pi-Pi**: Val 62 **Pi-alkyl**: Val 62B **Van der Waals**: Leu 35, Ala 60
7	Abrusogenin	−26.39	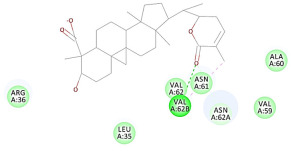	**H-bond**: Asn 62A, Val 62B **Pi-alkyl**: Val 62B **Van der waal**: Leu 35, Arg 36, Ala 60, Asn 61
8	Alloin	−24.93	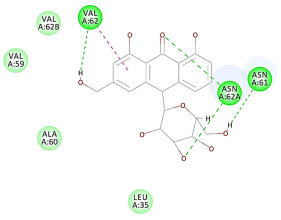	**H-bond**: Asn 61, Val 62, Asn 62A(×2) **Pi-amide**: Val 62 **Van der Waals**: Leu 35, Val 59, Ala 60, Val 62B
9	Avenanthramide A	−24.18	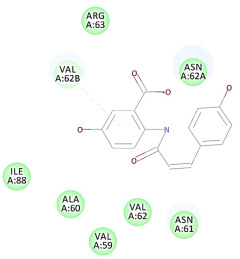	**H-bond**: Val 62B **Van der Waals**: Val 59, Ala 60, Asn 61, Val 62, Asn 62A, Arg 63, Ile 88
10	Nimbin	−22.68	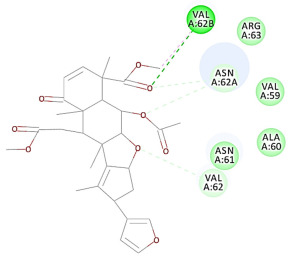	**H-bond**: Val 62, Asn 62A(×2), Val 62B **Pi-Alkyl**: Val 62B **Van der Waals**: Val 59, Ala 60, Asn 61, Arg 63
11	Eucryphin	−22.47	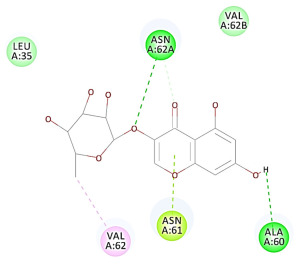	**H-bond**: Ala 60, Asn 62A **Pi-lone pair**: Asn 61 **Pi-alkyl**: Val 62 **Van der Waals**: Leu 35, Val 62B
12	Quercetin	−20.25	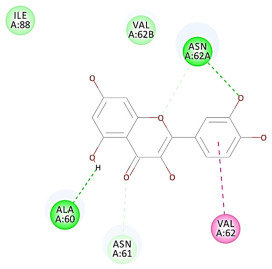	**H-bond**: Ala 60, Asn 61, Asn 62A **Pi-amide**: Val 62 **Van der Waals**: Val 62B, Ile 88
13	Shikonin	−19.80	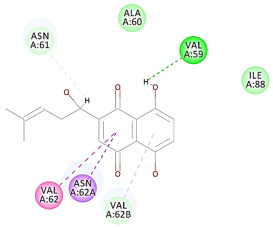	**H-bond**: Val 59, Asn 61, Val 62B **Pi-sigma**: Asn 62A **Pi-amide**: Val 62 **Van der Waals**: Ala 60, Ile 88
14	Bufadienolide	−18.71	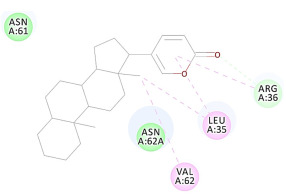	**H-bond**: Arg 36 **Pi-alkyl**: Leu 35(×2), Val 62 **Van der Waals**: Asn 61, Asn 62A
15	Genistein	−18.31	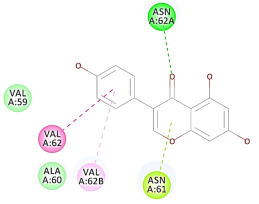	**H-bond**: Asn 62A **Pi-lone pair**: Asn 61 **Pi-amide**: Val 62 **Pi-alkyl**: Val 62B **Van der Waals**: Val 59, Ala 60
16	Lupeol	−18.19	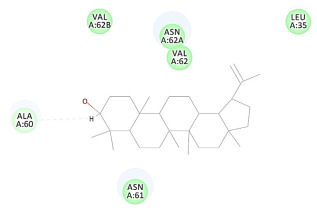	**H-bond**: Ala 60 **Van der waal**: Leu 35, Asn 61, Val 62, Asn 62A, Val 62B
17	Adiantone	−17.99	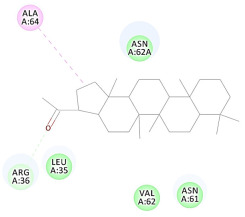	**H-bond**: Arg 36 **Pi-alkyl**: Ala 64 **Van der Waals**: Leu 35, Asn 61, Val 62, Asn 62A
18	Solaniol	−17.44	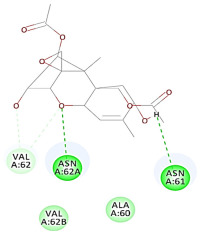	**H-bond**: Asn 61, Asn 62A, Val 62 **Van der Waals**: Ala 60, Val 62B
19	*N*-acetyl cysteine	−17.25	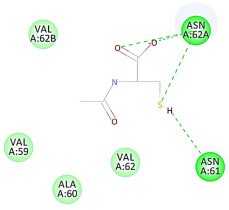	**H-bond**: Asn 61, Asn 62A (×3) **Van der Waals**: Val 59, Ala 60, Val 62, Val 62B
20	Berberine	−16.59	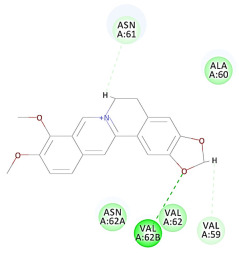	**H-bond**: Val 59, Asn 61, Val 62B **Van der Waals**: Ala 60, Val 62, Asn 62A
21	Alliin	−15.63	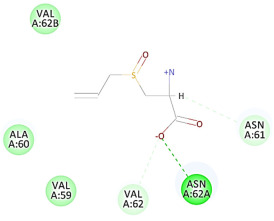	**H-bond**: Asn 61, Val 62, Asn 62A **Van der Waals**: Val 59, Ala 60, Val 62B
22	*S*-methyl-L-cysteine	−14.29	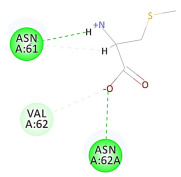	**H-bond**: Asn 61, Asn 62A, Val 62
23	E-caryophyllene	−11.78	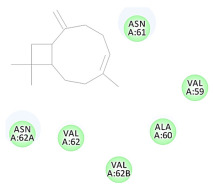	**Van der Waals**: Val 59, Ala 60, Asn 61, Val 62, Asn 62A, Val 62B
24	Limonene	−10.60	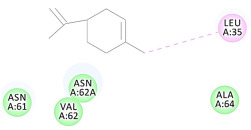	**Pi-alkyl**: Leu 35 **Van der Waals**: Asn 61, Val 62, Asn 62A, Ala 64

* Color reference: green dotted line indicates H-bond; faint green dotted line; indicates van der Waals interaction; lemon green dotted line indicates Pi-lone interaction; orange dotted line indicates attractive charge; dark purple dotted line indicates Pi-sigma bond; medium purple dotted line indicates Pi-amide bond; light purple dotted line indicates Pi-alkyl bond; pink dotted line indicates Pi-Pi bond.

## Data Availability

All data are provided in this review article.

## List of Abbreviations

DNA	Deoxyribonucleic acid
UV	Ultraviolet radiation
NF-κB	Nuclear factor-kappa enhancer binding protein
TNF-α	Tumor necrosis factor alpha
C3b	Complement component 3
NO	Nitric oxides
IL-1β	Interleukin 1 beta
TXB2	Thromboxane B2
COX-2	Cyclooxygenase-2
FDA	Food and Drug Administration
BC	Bacterial cellulose
PC	Plant cellulose
NK	Natural killer
HLE	Human leukocyte elastase
